# Estimating Prevalence of Overweight or Obese Children and Adolescents in Small Geographic Areas Using Publicly Available Data

**DOI:** 10.5888/pcd12.140229

**Published:** 2015-03-12

**Authors:** Carlo Davila-Payan, Michael DeGuzman, Kevin Johnson, Nicoleta Serban, Julie Swann

**Affiliations:** Author Affiliations: Carlo Davila-Payan, Kevin Johnson, Nicoleta Serban, Georgia Institute of Technology, Atlanta, Georgia; Michael DeGuzman, Columbia University Medical Center, New York, New York.

## Abstract

**Introduction:**

Interventions for pediatric obesity can be geographically targeted if high-risk populations can be identified. We developed an approach to estimate the percentage of overweight or obese children aged 2 to 17 years in small geographic areas using publicly available data. We piloted our approach for Georgia.

**Methods:**

We created a logistic regression model to estimate the individual probability of high body mass index (BMI), given data on the characteristics of the survey participants. We combined the regression model with a simulation to sample subpopulations and obtain prevalence estimates. The models used information from the 2001–2010 National Health and Nutrition Examination Survey, the 2010 Census, and the 2010 American Community Survey. We validated our results by comparing 1) estimates for adults in Georgia produced by using our approach with estimates from the Centers for Disease Control and Prevention (CDC) and 2) estimates for children in Arkansas produced by using our approach with school examination data. We generated prevalence estimates for census tracts in Georgia and prioritized areas for interventions.

**Results:**

In DeKalb County, the mean prevalence among census tracts varied from 27% to 40%. For adults, the median difference between our estimates and CDC estimates was 1.3 percentage points; for Arkansas children, the median difference between our estimates and examination-based estimates data was 1.7 percentage points.

**Conclusion:**

Prevalence estimates for census tracts can be different from estimates for the county, so small-area estimates are crucial for designing effective interventions. Our approach validates well against external data, and it can be a relevant aid for planning local interventions for children.

## Introduction

Obesity is considered an urgent health challenge and a winnable battle by the Centers for Disease Control and Prevention (CDC) (1). Because overweight or obese children are at a higher risk than normal-weight children for health problems, they are a target for intervention (2). There is evidence of disparities in pediatric obesity; Bethell et al (3) studied differences in obesity rates by race/ethnicity, insurance, and income and found within- and across-state disparities. Each of these factors can vary significantly across a city or county, so identifying small geographic areas with children at greatest risk for high body mass index (BMI) can be helpful in delivering cost-effective interventions. 

BMI data are obtained through direct measurement or self-reported survey data. Direct measurement results in more accurate data but is a more challenging and costly method; self-reporting results in inaccuracies and is generally biased among children younger than 12 years (4,5). Some cities or states began initiatives to measure height and weight in schools, but these systemic efforts are practiced in only a few places in the United States, such as Arkansas (6) and New York City (7). 

Approaches to estimating the prevalence of health conditions in small geographic areas are not new. Some researchers address uncertainty by using Bayesian approaches, which assume knowledge of the behavior being estimated (eg, psychiatry [8], hip and knee replacement [9]). Choy et al stressed the relevance of using publicly available information and presented an estimation method for an infectious disease but did not estimate variability (10). Methods for estimating the prevalence of adult obesity in small areas (11–14) cannot be easily applied for estimating prevalence among children. Using nonpublic data sets, Zhang et al (15) estimated the prevalence of obesity among American youths aged 10 to 17; they did not estimate the prevalence among younger children or the prevalence of overweight and obesity combined. Finally, to our knowledge, none of the previous studies described the prevalence of obesity among populations younger than 10 years or validated their estimates by comparing them with external measurement data.

Our objective was to describe a method that can be used to provide baseline estimates of the prevalence of children and adolescents with high BMI (either overweight and obese or obese only) at the census-tract level. We used US Census data and direct measurement data from the National Health and Nutrition Examination Survey (NHANES), and we piloted the method in Georgia’s 159 counties. The development of our approach originally responded to the need of a large health care provider to geographically target a large-scale campaign to reduce high BMI among children in Georgia. The same method can be applied to generate baseline estimates at other geographic levels, and using publicly available data makes our method easy and cost-effective to replicate.

## Methods

Our study used data on children and adolescents aged 2 to 17 years who were either overweight (BMI at or above the 85th percentile and lower than the 95th percentile for children of the same age and sex) or obese (BMI at or above the 95th percentile for children of the same age and sex) (16,17). We developed a model for predicting the probability of an individual child having high BMI using data from continuous NHANES surveys (2001–2010). We used R statistical software (18–20) to develop the model, generate samples, and map results. We created a simulation using C++ software (Intel Corporation) and data from the 2010 US Census to obtain prevalence estimates.

### Logistic regression model

In fitting the logistic model, we estimated Pr(*Y* = 1|*X*), where *Y* is the binary response and *X* is the vector of covariates.

#### Derivation of the dependent variable *Y*

We calculated BMI as a person’s weight in kilograms divided by the square of the person’s height in meters. The BMI-for-age charts adopted by the CDC in 2000 define children’s population percentiles by sex (16). We used charts for each age; 1 study (21) found that the BMI-for-age metric had less predictive power for children aged 3 to 5 years than it had for older children but did not invalidate BMI as a metric for obesity in very young children. We defined high BMI as a BMI in the 85th percentile or more (overweight or obese), which agrees with previous literature and CDC guidelines. We applied similar approaches for BMI values at or above the 95th percentile (obese).

#### Model Covariates: *X*

On the basis of previous findings (3,22), we used covariates related to socioeconomic and demographic status and potentially related to high BMI that are used in NHANES, the 2010 Census, and the American Community Survey (ACS). The variables considered were sex, race/ethnicity, age in months, education level of the household representative (level 1, <9th grade; 2, 9th–11th grade; 3, high school graduation or equivalent; 4, some college; 5, college graduate or above), household size (2 to ≥7 people), and family income or family poverty level (using the thresholds from ACS tables). Race/ethnicity and sex were treated as categorical variables.

#### Implementation of the logistic regression model 

The main logistic regression model included 6 variables: 3 binary variables for race/ethnicity (Hispanic, non-Hispanic black, non-Hispanic white, and other non-Hispanic), age of child in months, household size, and education level of household representative. The reference group was non-Hispanic white, a household size of 2 people, a child aged 2 years, and less than a 9th grade education.

We linearly scaled all variables into a [0,1] interval for numerical stability and comparison across covariates. We selected the covariates using backward stepwise variable elimination. The fitted logistic regression provided estimates for the conditional distribution of *Y*|*X*. We used bootstrap resampling to obtain realizations of the empirical distribution of the regression coefficients. 

### Simulation model

#### Generating virtual populations in geographic areas

We obtained demographic and socioeconomic data on census tracts from the US Census Bureau. Data on the distribution of the covariates were used to generate a virtual population of a geographic area. To more precisely characterize the population in a geographic area, we considered the interdependence of some population characteristics. We used multiple tables provided by the Census Bureau, each stratified by race and ethnicity (PCT20 and QTP2 from the 2010 Census for household size and age, respectively; B15001 from the 2010 ACS 5-year estimates for education).

#### Linking the individual high-BMI regression model to small-area-level data 

For each virtual individual *j* we estimated Pr(*Y* = 1*|X* = *Y_j_* ^*^) by sampling from the empirical distribution of the regression coefficients and evaluating the logistic probability function with the characteristics of the virtual individual. We use this probability to simulate *Y_j_* ^*^, the binary weight status of the individual. The prevalence estimate of high BMI is then

Ρ^=ΣjΒ=1Yj*Β

where *B* is the population count in the geographic area. We repeated the simulation 1,000 times and obtained the standard deviation of the estimate. This simulation model allowed for variations due to model estimation and individual randomness. It took less than an hour to produce the estimates for Georgia.

### Identification of priority areas

When limited resources are assigned to improve an overall system indicator, a common approach is to allocate most resources to where the largest overall benefit is obtained. This idea is known as the Pareto principle (23). In our context, 2 indicators were relevant for classifying small areas by priority. The first indicator was the estimated baseline prevalence for the area. The second was the estimated number of children with a high BMI (the estimated prevalence × the population of children) in each area. We used the Pareto principle to select priority areas for intervention — ie, areas with the largest number of children with high BMI. We assigned priority to the counties that accounted for approximately 77% of the total population of children with high BMI.

### Model validation

We developed 3 analyses to validate our modeling approach. One, we modeled the population aged 10 to 17 years in Georgia and compared our state-level outcomes to the state-level prevalence estimates of the 2007 National Survey of Children’s Health (24). Two, we modeled the population of adults and compared our county-level results with CDC’s 2007 county-level obesity estimates for Georgia (25). Three, we modeled obesity among children aged 5 to 17 years in Arkansas by county and compared our data with the 2010–2011 school measurements in that state (6). For the adult validation model, additional variables were added for a better fit, and for the Arkansas validation model, income was added to capture variation in demographics in that state.

## Results

### Logistic regression model

In Georgia, non-Hispanic black children and Hispanic children were more likely to have high BMI than non-Hispanic white children, and other non-Hispanics were less likely (Table 1). The probability of high BMI increased with age. The probability of high BMI decreased as the education level of the household representative increased. The probability of a high BMI also decreased with household size. For predicting obesity only, the model variables were the same, but the coefficients were different (Table 1). 

**Table 1:** Results of Logistic Regression Model for Overweight or Obese and Obese Only, Children and Adolescents Aged 2 to 17 Years, Georgia, 2000–2010^a^^,^ b

Covariate	Adjusted Coefficient Estimate (SE) [*P* Value]	
	Overweight or Obese	Obese Only
Intercept	−0.45 (0.12) [<.001]	−1.25 (0.14 [<.001]
Non-Hispanic black	0.24 (0.06) [<.001]	0.33 (0.07) [<.001]
Non-Hispanic other	−0.22 (0.10) [.02]	−0.28 (0.11) [.01]
Hispanic	0.34 (0.06) [<.001]	0.36 (0.09) [<.001]
Education level of household representative	−0.60 (0.09) [<.001]	−0.76 (0.12) [<.001]
Household size	−0.66 (0.10) [<.001]	−0.66 (0.12) [<.001]
Age, mo	0.68 (0.06) [<.001]	0.69 (0.14) [<.001]

Abbreviation: SE, standard error.

a Values of covariates were scaled to a [0,1] interval before regression.

b *P* value of the model calculated from Wald *F* and Wald χ^2^ tests was <.001.

### Simulation model

Among a sample of census tracts in Georgia (Table 2), the prevalence of high BMI was significantly lower in census tract no. 20300 (27.4%) than in neighboring census tract no. 20600 (36.4%) in DeKalb County. One explanation is that the tracts had different socioeconomic and demographic characteristics, except for household size (Table 3).

**Table 2:** Examples of Census-Tract Prevalence Estimates of Overweight or Obese Children and Adolescents Aged 2 to 17 Years, Georgia, 2000–2010

County	Tract No.	Mean % (Standard Deviation)
Cobb	031110	33.5 (2.85)
	031112	30.8 (1.51)
	031205	32.9 (1.75)
	031206	31.7 (1.29)
DeKalb	020300	27.4 (1.70)
	020400	28.2 (2.43)
	020500	38.3 (2.86)
	020600	36.4 (2.26)
Fulton	000400	30.6 (1.46)
	000500	30.6 (2.11)
	000600	32.8 (3.54)
	000700	36.6 (3.68)
Muscogee	000900	35.1 (2.10)
	001000	32.6 (1.87)
	001100	28.9 (1.79)
	001200	32.5 (1.95)

**Table 3:** Characteristics of Two Sample Census Tracts in Georgia, 2000–2010

Characteristic	Census Tract No. 20300	Census Tract No. 20600
**Race/ethnicity, %**		
Non-Hispanic white	84	8
Non-Hispanic black	4	89
Hispanic	4	1
Other non-Hispanic	8	3
**Household size, no. of people**	3.0	3.0
**Education level of household representative^a^**	4.2	3.7
**Average age of children, y**	7.5	8.7

a Education level of the household representative: level 1, <9th grade; 2, 9th–11th grade; 3, high school graduation or equivalent; 4, some college; 5, college graduate or above.

The estimated prevalence of high BMI at the census-tract level in Georgia varied from 26% to 42%. Counties with the greatest variability in census-tract prevalence estimates also tended to be the counties with the largest populations (Supplementary Figure [Appendix]). In Fulton County, for example, the prevalence of a high BMI by census tract ranged from 26% to 41%. The prevalence by county varied from 31% to 40% (Supplemental Table 4 [Appendix]). According to the Census Bureau, census tracts are generally defined according to observable characteristics and features, whereas counties are usually larger and may include areas with more diverse characteristics (26); these differences may explain the differences in prevalence ranges. 

**Figure 1:**
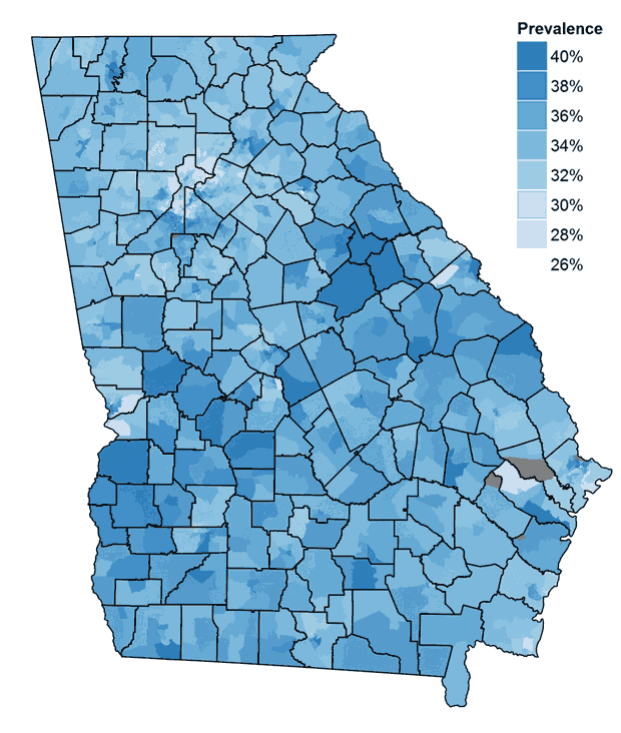
Prevalence estimates of a high body mass index among children and adolescents aged 10 to 17 in census tracts in Georgia in 2010. The gray areas indicate areas with no population (eg, airports, parks).

The prevalence of high BMI was low in the northern part of Atlanta, whereas prevalence was higher in some areas of the eastern, western, and southern parts of the city (Figure 1). The correlation at the census-tract level between the prevalence estimates for obesity and prevalence estimates for overweight or obesity was 0.972. 

### Identification of priority areas

**Figure 2:**
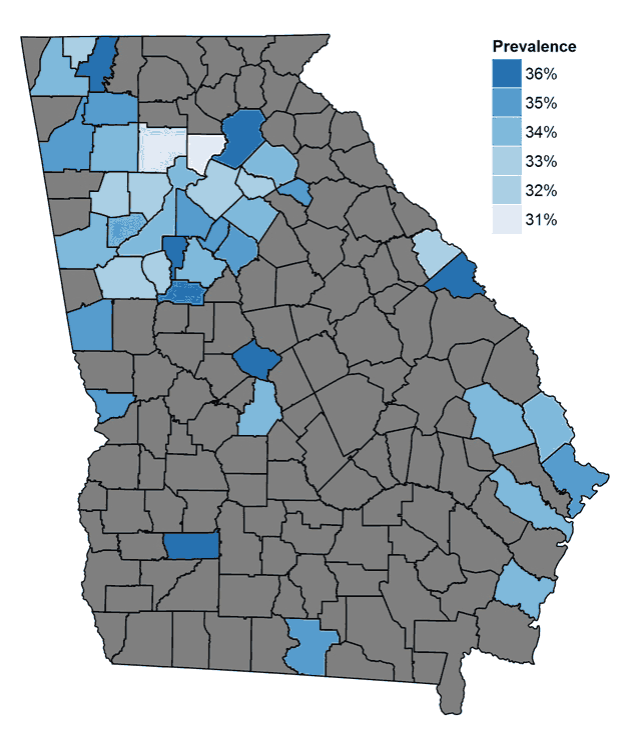
The prevalence of high body mass index (BMI) in the 25% of counties (n = 39) in Georgia with the greatest estimated number of children and adolescents with high BMI. Of all children and adolescents with high BMI in Georgia, 77% reside in these 39 counties. These counties are strongly correlated with population centers. Gray areas indicate the other 75% of counties. **Table:** 

Approximately 77% of children with high BMI resided in 39 counties (25% of counties) in Georgia (Figure 2). Areas of high BMI included densely populated areas, such as metropolitan Atlanta, smaller cities such as Augusta, Macon, Savannah, and Rome, as well as rural areas. 

County	County No.	Estimated Prevalence of High Body Mass Index, %	Estimated Number of Children with High Body Mass Index
Gwinnett	13135	33.1	210,789
Fulton	13121	33.4	194,766
Cobb	13067	32.9	157,838
Dekalb	13089	34.6	144,462
Clayton	13063	35.9	66,450
Henry	13151	33.6	54,486
Chatham	13051	34.5	52,468
Cherokee	13057	31.7	53,061
Hall	13139	35.3	44,691
Richmond	13245	35.5	43,417
Muscogee	13215	34.9	42,900
Forsyth	13117	30.5	48,578
Paulding	13223	32.8	38,912
Bibb	13021	35.8	35,524
Douglas	13097	34.3	33,807
Houston	13153	33.7	33,529
Coweta	13077	33.0	31,206
Columbia	13073	32.2	30,681
Whitfield	13313	36.7	26,179
Newton	13217	34.4	26,057
Fayette	13113	32.6	26,414
Carroll	13045	34.0	25,047
Bartow	13015	34.1	24,150
Lowndes	13185	34.4	23,613
Dougherty	13095	36.4	21,498
Floyd	13115	34.8	21,004
Rockdale	13247	35.1	20,669
Walton	13297	33.5	20,417
Clarke	13059	35.0	17,569
Glynn	13127	34.2	17,119
Barrow	13013	33.0	17,340
Liberty	13179	33.8	16,466
Troup	13285	34.8	15,975
Spalding	13255	35.3	14,403
Walker	13295	33.7	14,607
Jackson	13157	33.4	14,410
Catoosa	13047	32.6	14,422
Gordon	13129	34.9	13,281
Effingham	13103	33.3	13,551
Bulloch	13031	33.8	12,779

### Model validation

Our baseline prevalence estimate of high BMI among children aged 10 to 17 years in Georgia was 37.5%. The 2007 state-level estimate in the National Survey of Children’s Health was 37.3% (95% confidence interval, 31.7%–42.9%) (24).

When we compared our county-level estimates for adults in Georgia with CDC’s estimates (25), we obtained a 0.92 spatial correlation; the median difference between counties in the 2 sets of estimates was less than 1.3 percentage points. When we compared our county-level estimates of overweight or obesity among children aged 5 to 17 years in Arkansas with the 2010–2011 county measurements (6), we obtained a spatial correlation of 0.77; the median difference between counties was 1.7 percentage points. Supplementary Table 5 and Table 6 (Appendix) provide details on the adult and Arkansas models.

## Discussion

Results are not surprising for the race/ethnicity variables in the model predicting an individual’s probability of high BMI, because they are consistent with prior research on the subject (27). The variable of income was not selected in the best-fitting models for Georgia, but alternative models were possible with that variable. There is no obvious explanation for why household size affects the probability of high BMI, but this question is worth future study. Other factors associated with high BMI in adults are cardiovascular diseases and smoking (28); for children, we considered alternative models with a variable to indicate whether anyone in the child’s home smokes, but we found no significant improvement in the regression model; this factor may have been captured indirectly by other variables.

The selection of independent variables for a model depends on the estimate or region being studied. For our analysis of Georgia, the great majority of the population comprised 3 main racial/ethnic groups: black, Hispanic, and white. In Arkansas, race/ethnicity was more homogeneous than in Georgia, so our model there included income. If our approach is to be used in any other state, the selection of the model variables should match the composition of that state’s population.

Validations indicated that our modeling methodology can provide reasonable estimates, with high correlation with reference values and good accuracy. However, the model-based estimates had smaller ranges than the validation data. External data were not available to validate prevalence estimates for children younger than 5 years.

Data showing differences in prevalence estimates among census tracts in a single county support the importance of generating estimates for small areas. Because the populations of census tracts and counties in our study were heterogeneous in several respects, small-area estimations provided better information than county-based measures. Small-area estimations can help to target interventions aimed at high BMI and can also be used for targeting interventions for other public health problems. The findings described here, including the strategy for identifying priority areas, were used by a health provider in Georgia to prioritize interventions for children with high BMI throughout the state. 

Small-area estimates are useful for informing intervention strategies, but they are more difficult to use for evaluating interventions. One reason for the difficulty is that data may not change quickly enough to drive new estimates. This is especially true for a condition like obesity, the data for which change slowly. On the other hand, small-area estimates for diseases such as human immunodeficiency virus may be more dynamic, especially if the estimates are able to incorporate local information.

Our study has several limitations. Because we included individual-level variables only and not local context (13), our results could be over-smoothed and could underestimate geographic variations in a geographic unit such as a county. Many factors related to high BMI among children may be specific to a geographic area, and data on these factors cannot be entirely captured without local sampling (29). Our model can capture precisely only those interactions among population variables that are publicly available in the US Census, potentially introducing bias to the estimates when assuming partial independence among some of the input variables. Additionally, complete information is not available on some small groups in census tracts, adding levels of approximation to our estimates in smaller areas. Our model is limited to the validity of the data used. For example, Eto et al (30) found that BMI had low sensitivity but high specificity for predicting obesity in children aged 3 to 5 years. The ACS provides yearly estimates for every variable used in our model; these estimates can be updated annually. The NHANES data used to develop our model were from 2001 through 2010, which ignores the temporal trends in pediatric obesity; however, a recent study found childhood obesity has not considerably changed during the past decade (27).

We presented a cost-effective and sound method for estimating the prevalence of obesity in small geographic areas. The method is based on publicly available data and can be used in the absence of local surveillance data; it can be used to inform interventions for children with high BMI. The prevalence estimates generated by our model served to build maps of baseline estimation of obesity prevalence in Georgia; used with appropriate caution, the model can help build baseline estimations for other states or diseases without publicly available small-area estimates. To the best of our knowledge, we are the first to generate obesity estimates for children younger than 10 years and the first to validate the accuracy of small-area estimates of childhood obesity prevalence with external measurement data. We plan to make our code publicly available online. Future improvements to our model are to use prevalence estimates of adult and youth overweight and obesity for larger areas (counties or states) to inform prevalence estimates for small areas, such as census tracts. 

## Figures and Tables

**Supplementary Figure:**
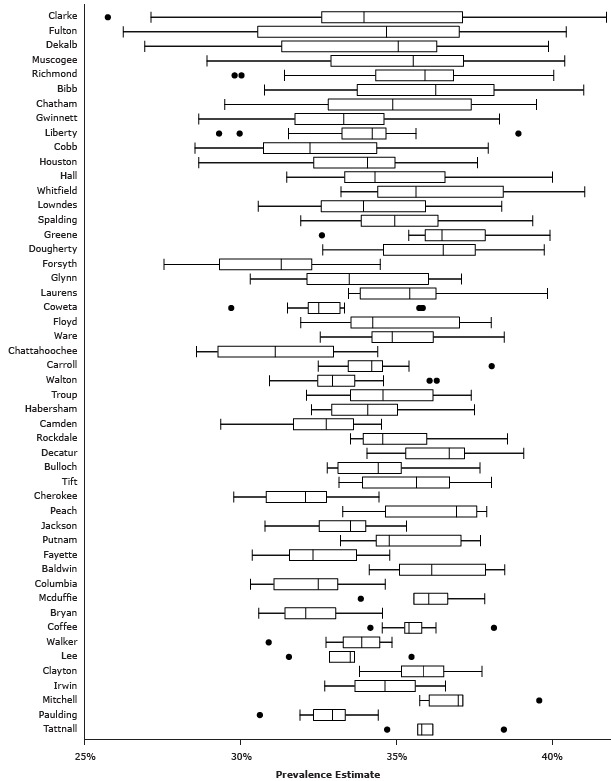
Prevalence-estimate ranges across census tracts for the 50 counties in Georgia with the largest ranges. The vertical line in the middle of each box indicates the median; the left and right borders of the box represent the 25th and 75th percentiles, respectively; the right and left whiskers mark the minimum and maximum, respectively; the dots represent outliers as determined by 1.5 times the difference between the 25th and 75th percentiles. **Table:** 

County	Minimum	25th Percentile	Median	75th Percentile	Maximum
Clarke	25.8	32.6	34.0	37.1	41.7
Fulton	26.3	30.6	34.7	37.0	40.4
Dekalb	26.9	31.3	35.1	36.3	39.9
Muscogee	28.9	32.9	35.5	37.1	40.4
Richmond	29.8	34.3	35.9	36.8	40.0
Bibb	30.8	33.8	36.3	38.1	41.0
Chatham	29.5	32.8	34.9	37.4	39.5
Gwinnett	28.7	31.8	33.3	34.6	38.3
Liberty	29.3	33.3	34.2	34.7	38.9
Cobb	28.6	30.7	32.2	34.4	37.9
Houston	28.7	32.4	34.1	35.0	37.6
Hall	31.5	33.3	34.3	36.6	40.0
Whitfield	33.2	34.4	35.6	38.4	41.0
Lowndes	30.6	32.6	33.9	35.9	38.4
Spalding	31.9	33.9	35.0	36.3	39.4
Greene	32.6	35.9	36.5	37.8	39.9
Dougherty	32.6	34.6	36.5	37.5	39.7
Forsyth	27.6	29.3	31.3	32.3	34.5
Glynn	30.3	32.1	33.5	36.0	37.1
Laurens	33.5	33.8	35.4	36.3	39.8
Coweta	29.7	32.2	32.5	33.2	35.8
Floyd	31.9	33.5	34.2	37.0	38.0
Ware	32.6	34.2	34.9	36.2	38.5
Chattahoochee	28.6	29.3	31.1	33.0	34.4
Carroll	32.5	33.5	34.2	34.6	38.1
Walton	30.9	32.5	33.0	33.7	36.3
Troup	32.1	33.5	34.6	36.2	37.4
Habersham	32.3	32.9	34.1	35.0	37.5
Camden	29.4	31.7	32.8	33.6	34.5
Rockdale	33.5	33.9	34.6	36.0	38.6
Decatur	34.1	35.3	36.7	37.2	39.1
Bulloch	32.8	33.1	34.4	35.2	37.7
Tift	33.2	33.9	35.6	36.7	38.0
Cherokee	29.8	30.8	32.1	32.8	34.4
Peach	33.3	34.7	36.9	37.6	37.9
Jackson	30.8	32.5	33.5	34.0	35.3
Putnam	33.2	34.4	34.8	37.1	37.7
Fayette	30.4	31.6	32.3	33.7	34.8
Baldwin	34.1	35.1	36.1	37.9	38.5
Columbia	30.3	31.1	32.5	33.1	34.6
McDuffie	33.9	35.6	36.0	36.6	37.8
Bryan	30.6	31.4	32.1	33.1	34.6
Coffee	34.2	35.3	35.4	35.8	38.1
Walker	30.9	33.3	33.9	34.5	34.9
Lee	31.6	32.9	33.5	33.7	35.5
Clayton	33.8	35.2	35.9	36.5	37.7
Irwin	32.7	33.7	34.6	35.6	36.6
Mitchell	35.8	36.1	37.0	37.1	39.6
Paulding	30.6	32.4	33.0	33.4	34.4
Tattnall	34.7	35.7	35.8	36.2	38.4

## Supplementary Table 4. County-Level Prevalence Estimates for Children Aged 2 to 17 Years in Georgia Who Are Overweight or Obese or Obese Only, 2000–2010

**Table:**

County	County No.	Obese, %	Obese or Overweight, %
Appling	13001	19.5	35.7
Atkinson	13003	21.1	37.6
Bacon	13005	19.4	35.3
Baker	13007	20.7	36.2
Baldwin	13009	20.1	35.9
Banks	13011	18.3	34.5
Barrow	13013	17.6	33.0
Bartow	13015	18.3	34.1
Ben Hill	13017	20.4	36.4
Berrien	13019	19.0	35.0
Bibb	13021	20.3	35.8
Bleckley	13023	19.9	36.1
Brantley	13025	18.0	34.2
Brooks	13027	20.2	36.0
Bryan	13029	17.1	32.3
Bulloch	13031	18.0	33.9
Burke	13033	20.9	36.5
Butts	13035	19.3	35.0
Calhoun	13037	21.5	37.3
Camden	13039	17.6	32.8
Candler	13043	19.6	35.8
Carroll	13045	18.2	34.0
Catoosa	13047	17.0	32.6
Charlton	13049	19.1	35.2
Chatham	13051	19.1	34.5
Chattahoochee	13053	16.3	30.7
Chattooga	13055	18.6	34.6
Cherokee	13057	16.5	31.7
Clarke	13059	19.4	35.0
Clay	13061	22.3	38.0
Clayton	13063	20.5	35.9
Clinch	13065	19.7	35.3
Cobb	13067	17.5	32.9
Coffee	13069	20.0	35.8
Colquitt	13071	20.2	36.4
Columbia	13073	16.8	32.2
Cook	13075	19.6	35.6
Coweta	13077	17.6	33.0
Crawford	13079	19.0	34.9
Crisp	13081	21.2	36.9
Dade	13083	17.5	33.4
Dawson	13085	17.2	33.2
Decatur	13087	20.6	36.4
Dekalb	13089	19.5	34.6
Dodge	13091	20.2	36.0
Dooly	13093	22.1	38.0
Dougherty	13095	20.8	36.4
Douglas	13097	18.9	34.3
Early	13099	21.0	36.6
Echols	13101	19.5	36.0
Effingham	13103	17.7	33.3
Elbert	13105	20.7	36.8
Emanuel	13107	20.3	36.1
Evans	13109	20.4	36.3
Fannin	13111	17.9	34.2
Fayette	13113	17.1	32.6
Floyd	13115	18.9	34.8
Forsyth	13117	15.5	30.5
Franklin	13119	18.5	34.5
Fulton	13121	18.2	33.4
Gilmer	13123	18.8	35.1
Glascock	13125	18.6	34.4
Glynn	13127	18.6	34.2
Gordon	13129	18.6	34.9
Grady	13131	20.2	36.0
Greene	13133	21.3	37.5
Gwinnett	13135	18.0	33.1
Habersham	13137	18.6	34.6
Hall	13139	19.2	35.3
Hancock	13141	23.8	39.7
Haralson	13143	18.0	34.1
Harris	13145	17.3	33.1
Hart	13147	19.1	35.1
Heard	13149	18.4	34.5
Henry	13151	18.4	33.6
Houston	13153	18.2	33.7
Irwin	13155	19.7	35.6
Jackson	13157	17.6	33.4
Jasper	13159	18.8	34.4
Jeff Davis	13161	19.3	35.4
Jefferson	13163	21.5	37.3
Jenkins	13165	20.5	36.4
Johnson	13167	20.4	36.5
Jones	13169	18.4	33.9
Lamar	13171	19.3	35.1
Lanier	13173	18.9	34.3
Laurens	13175	19.8	35.5
Lee	13177	17.8	33.3
Liberty	13179	18.4	33.8
Lincoln	13181	20.0	36.0
Long	13183	18.9	34.7
Lowndes	13185	18.7	34.4
Lumpkin	13187	17.1	33.3
Macon	13189	20.8	36.6
Madison	13191	20.4	36.2
Marion	13193	21.8	37.9
McDuffie	13195	18.6	34.7
McIntosh	13197	20.5	36.4
Meriwether	13199	20.4	36.4
Miller	13201	19.8	35.6
Mitchell	13205	21.3	37.0
Monroe	13207	18.7	34.7
Montgomery	13209	19.1	34.9
Morgan	13211	18.7	34.5
Murray	13213	19.0	35.4
Muscogee	13215	19.5	34.9
Newton	13217	19.1	34.4
Oconee	13219	16.1	31.4
Oglethorpe	13221	18.8	34.6
Paulding	13223	17.3	32.8
Peach	13225	19.9	35.9
Pickens	13227	17.2	33.2
Pierce	13229	18.3	34.5
Pike	13231	17.5	33.1
Polk	13233	19.3	35.1
Pulaski	13235	20.0	35.6
Putnam	13237	19.4	35.5
Quitman	13239	22.1	38.0
Rabun	13241	18.3	34.5
Randolph	13243	22.3	38.1
Richmond	13245	20.1	35.5
Rockdale	13247	19.6	35.1
Schley	13249	19.4	35.1
Screven	13251	21.0	37.1
Seminole	13253	20.5	36.4
Spalding	13255	19.6	35.3
Stephens	13257	18.4	34.5
Stewart	13259	22.8	38.5
Sumter	13261	21.3	37.0
Talbot	13263	22.2	38.0
Taliaferro	13265	23.8	39.8
Tattnall	13267	20.1	36.2
Taylor	13269	21.5	37.2
Telfair	13271	20.9	36.6
Terrell	13273	22.4	38.3
Thomas	13275	19.5	35.3
Tift	13277	19.9	35.7
Toombs	13279	19.6	35.4
Towns	13281	17.7	33.7
Treutlen	13283	20.0	35.7
Troup	13285	19.1	34.9
Turner	13287	20.7	36.5
Twiggs	13289	21.3	37.5
Union	13291	17.6	33.5
Upson	13293	19.7	35.7
Walker	13295	17.9	33.7
Walton	13297	18.1	33.5
Ware	13299	19.5	35.2
Warren	13301	23.1	38.8
Washington	13303	21.3	37.2
Wayne	13305	18.7	34.4
Webster	13307	20.5	35.5
Wheeler	13309	20.0	35.6
White	13311	17.4	33.2
Whitfield	13313	20.2	36.7
Wilcox	13315	20.3	36.2
Wilkes	13317	21.0	37.1
Wilkinson	13319	20.3	36.0
Worth	13321	20.1	35.8

## Supplementary Table 5. Results of Logistic Regression Model for Overweight or Obese Children Aged 5 to 17 Years Used for Arkansas Prevalence Estimates^a^

**Table:**

Variable	Adjusted Coefficient Estimate (SE)
Intercept	−1.136 (0.142)
Non-Hispanic black	0.268 (0.072)
Other non-Hispanic	−0.326 (0.103)
Hispanic	0.311 (0.088)
Education level of household representative	−0.628 (0.138)
Household size	−0.714 (0.119)
Age in months	0.732 (0.094)
Poverty ratio	−0.348 (0.110)

Abbreviation: SE, standard error.

^a^ Values of covariates were scaled to a [0,1] interval before regression.

## Supplementary Table 6. Adjusted Coefficient Estimates of the Logistic Regression Model Used for Adult Prevalence Estimates in Georgia^a^

**Table:**

Variable	Age Group, y					
	18–24	25–34	35–44	45–54	55–64	≥65
Intercept	−1.61	−1.4	−0.43	−0.36	−0.36	−0.5
Black non-Hispanic	0.54	0.78	0.44	0.42	0.35	0.51
Hispanic	0	0.3	0	0	0	0
Other non-Hispanic	0	0	−0.67	−0.67	−0.85	−1.08
Education level of household representative	−0.19	0.03	−0.12	−0.11	−0.09	−0.08
Household size	0	0	0	0.05	0	0
Age	0.14	0.04	0	0	0	−0.05
Sex	0.33	0.25	0	0	0.19	0
NHANES year	0	0.07	0.07	0	0	−0.13

Abbreviation: NHANES, National Health and Nutrition Examination Survey.

^a^ Values of covariates were scaled to a [0,1] interval before regression, and a new model was created for each age group listed.
